# Potential mediating role of self-concealment in the relationship between family resilience and stigma among women who have had induced abortions: a cross-sectional study

**DOI:** 10.3389/fpubh.2026.1877273

**Published:** 2026-07-01

**Authors:** Tingting Ruan, Yiwei Li, Xuyan Liu, Ying Yuan

**Affiliations:** Department of Gynecology, Deyang People's Hospital, Deyang, Sichuan, China

**Keywords:** abortion, induced, shame, social stigma, women

## Abstract

**Objectives:**

To explore the factors associated with stigma among women undergoing induced abortion in China and to investigate whether self-concealment plays a potential mediating role between family resilience and stigma.

**Methods:**

This was a cross-sectional study conducted between A total of 288 women who had induced abortions at a public hospital in Sichuan Province, China, filled out the Individual-Level Abortion Stigma scale, the family resilience scale, and the Self-Concealment Scale. To identify factors associated with stigma in women who have undergone induced abortions, independent sample *t*-tests or one-way ANOVA were employed. The bootstrapping technique was utilized to examine the potential mediating role of self-concealment.

**Results:**

This research indicated that the stigma score for women who underwent induced abortions was 50.12 ± 11.57. Stigma was potentially associated with factors including age (F = 4.28, *P* < 0.050). Marital status (*t* = 5.59, *P* < 0.001) and education level (F = 9.18, *P* < 0.001). Self-concealment showed a potential mediating role in the relationship between family resilience and stigma (indirect effect −0.09, 95.0% CI −0.13 to −0.05).

**Conclusions:**

Elevated stigma levels in women undergoing induced abortions may be associated with various sociodemographic factors. Self-concealment may serve as a potential mediator of the relationship between family resilience and stigma.

## 1 Introduction

Approximately 70 million women worldwide undergo induced abortions each year (1). In China, this figure reaches 10 million annually (2). Clinical practice often focuses on the physical damage to women undergoing induced abortions, while their psychological trauma is less frequently addressed. In addition, they may also be vulnerable to other psychological issues, including fear, feelings of inferiority, pessimism, hostility, and post-traumatic stress (3). Stigma is an independent risk factor that affects the mental health of women undergoing induced abortions (4). The stigma experienced by these women can impede access to high-quality care (5) and contribute to health inequality (6). Acknowledging the critical need to safeguard the health and welfare of women pursuing abortions, the World Health Organization has advocated for abortion care services that are free from stigma.

Stigma is defined as the holding of negative and biased attitudes toward individuals, including behaviors such as humiliation, feelings of inferiority, provision of inadequate information, disregard for the individual's ability to act responsibly, and reinforcement of cultural stereotype s (7). This encompasses self-stigma (lowered self-worth and stigma from adopting negative views), social stigma (facing discrimination from peers or relatives), and structural stigma (obstacles created by regional health and social service regulations) (8). Women who undergo induced abortions may feel guilt that stifles life (9) and may be judged, ridiculed, or opposed from others (4). Some women seeking abortion services may encounter apathy from medical personnel (10, 11). Furthermore, research indicates that medical professionals in certain faith-based health institutions are even less inclined to offer abortions or abortion referrals (12). These elements can greatly impact women's overall quality of life. Particularly in some Asia countries, there are traditional beliefs that childbearing is a pivotal life event, with most women holding the view that “passing on the family line” is their obligation and mission (13, 14). In such an environment, women who are forced to terminate a pregnancy through induced abortion may perceive it as a failure to fulfill their procreative obligation, leading to feelings of inferiority and self-stigma. They may also face social stigma and moral pressure, and in some cases, this can even lead to the breakdown of marital relationships between spouses (15, 16). In China, the majority of the population, especially parents, still hold a conservative attitude toward open sex culture, with prenuptial sex considered a disgrace and associated with immorality, and women undergoing induced abortion are easily stigmatized (17). Therefore, further research is needed to evaluate the stigma among women undergoing induced abortion under specific culture contexts.

Family resilience refers to the family's ability to resist or maintain its patterns when facing stressors and to recover from them (18). Existing studies suggest that family resilience may be associated with stigma, as increased family resilience appears to help alleviate negative psychological feelings (19). Moreover, a recent study among infertile women undergoing *in vitro* fertilization found that family resilience was significantly negatively associated with infertility stigma (20). Abortion is a sensitive and private topic, and many women are unwilling to discuss it with others or may actively conceal their experience of induced abortion, leaving them vulnerable to stigma (4). It has been proposed that family resilience can provide care and support to women who have abortions, potentially enabling them to actively confront and manage the various challenges that may arise following the procedure, thereby possibly mitigating the psychological burden and stigma associated with abortion (19). Based on these findings, family resilience may represent a key protective factor associated with stigma in women undergoing induced abortions, although direct empirical evidence in this specific population remains limited (20).

Self-concealment refers to the psychological tendency of people to deliberately hide their distress or unfavorable personal details from others (21). This tendency has been identified as being potentially predictive of the emergence of negative psychological problems (22, 23). Existing research has highlighted a significant relationship between self-concealment and the experience of stigma (24). Although self-concealment may provide a temporary buffer against stigma. In the long term, it can lead aborting women to focus on the perceived negative consequences of abortion on themselves and their families, thereby resulting in a substantial psychological burden (25). Moreover, self-concealment can hinder the timely receipt of support and guidance from others, potentially exacerbating individual psychological distress and social pressure, which may give rise to various psychological issues (22). Additionally, studies have linked individual family resilience with self-concealment, indicating that self-concealment may serve as a risk factor in relation to family resilience (26). However, the relationships among family resilience, self-concealment, and stigma in women who have had induced abortions remain unclear.

Despite the evidence outlined above, several important research gaps persist. First, although family resilience and self-concealment have been separately examined in relation to stigma in other populations, to our knowledge, no study to date has simultaneously investigated all three constructs in women who have undergone induced abortions. Second, the potential mediating mechanism of self-concealment between family resilience and stigma remains unexplored in this specific population. Third, existing studies have primarily focused on Western or general samples, limiting the generalizability of findings to Chinese cultural contexts where abortion-related stigma may be particularly pronounced due to traditional values regarding premarital sex and procreation (13, 14, 17). Therefore, the present study aims to (a) examine the factors associated with stigma among Chinese women who have had induced abortions, and (b) investigate whether self-concealment plays a potential mediating role in the relationship between family resilience and stigma.

**Figure 1:**
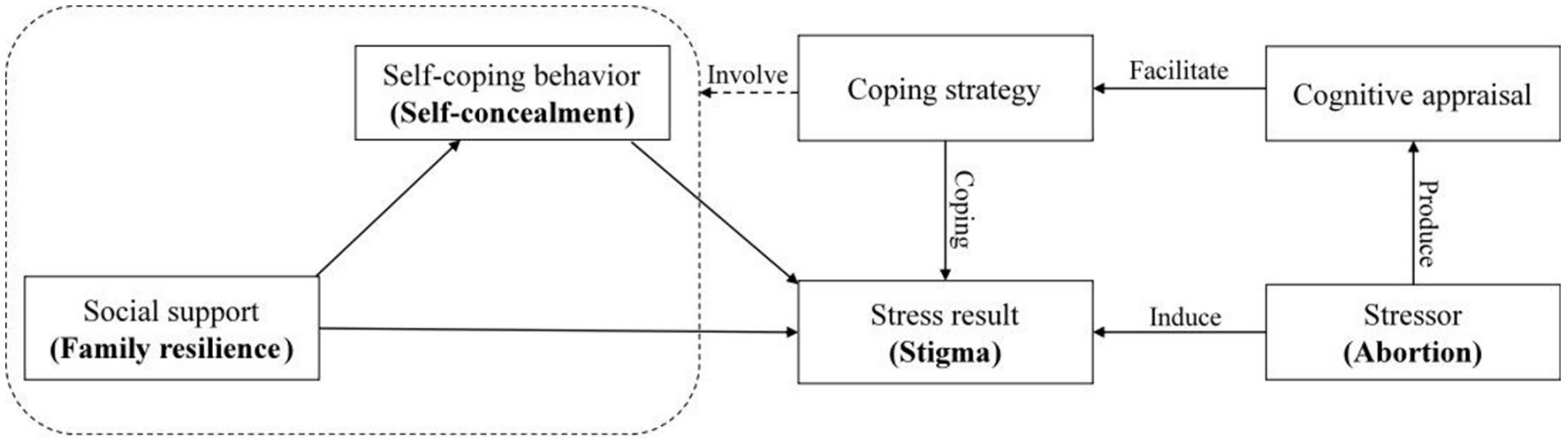
Theoretical model of this study.

Psychological stress theory refers to the individual's response to stressful events, which arises from the interaction of stressors and various other factors (27). According to this theory, the triggers of stress are typically life events. In the context of this study, induced abortion is considered as a stressor, leading to stigmatization as one of the outcomes of stress. Individuals differ in their cognitive appraisals of stressors, adopt varying coping strategies, such as the self-concealment behavior and utilize different forms of social support, including the extent of family resilience demonstrated in this study, to manage stress. Thus, theoretically, both family resilience and self-concealment may be correlated with stigma, and evidence from studies involving women who had abortions supports these relationships. In particular, women who undergo abortions might choose to conceal this information to prevent facing societal prejudice (19). However, robust family resilience could empower these women to fully engage family resources internally and externally to proactively address abortion-related challenges (28). In addition, according to McCubbin's family resilience model (29), a high level of family resilience may mitigate the tendency to employ negative coping strategies and, theoretically, could help reduce stigma by diminishing self-concealment. Preliminary empirical research has provided some support for this notion, indicating that women from more resilient families are more likely to express their vulnerability and distress to their families rather than conceal it (19). Figure 1 illustrates the conceptual framework of this research. Consequently, we propose that self-concealment may play a potential mediating role in the relationship between family resilience and stigma in women who have undergone abortions.

### 1.1 Hypotheses

Based on the theoretical framework and literature review, the following hypotheses were proposed: (1) Family resilience is negatively associated with stigma. (2) Self-concealment is positively associated with stigma. (3) Self-concealment plays a mediating role in the relationship between family resilience and stigma.

## 2 Materials and methods

### 2.1 Study design

This study was a cross-sectional design using convenience sampling method. Due to this cross-sectional design, causal relationships among family resilience, self-concealment, and stigma cannot be established, and the mediation analysis should be interpreted as exploratory and association-based.

### 2.2 Setting, participants, and procedure

This study recruited participants from one tertiary hospitals in Sichuan Province, China, between December 2024 to November 2025. With the consent of the hospitals' relevant departments, we initially screened the patient system of the induced abortion registry for eligibility. Eligible women were invited to join the study if they met these conditions: (1) they had an induced abortion over a week ago (30, 31); (2) their pregnancy was under 12 weeks; (3) they willingly agreed to participate. Individuals with mental illness, hearing impairment, language communication barriers, or other serious comorbidities were excluded from the study. Surveys were gathered via in-person interviews, and every participant gave informed consent prior to starting.

**Figure 2:**
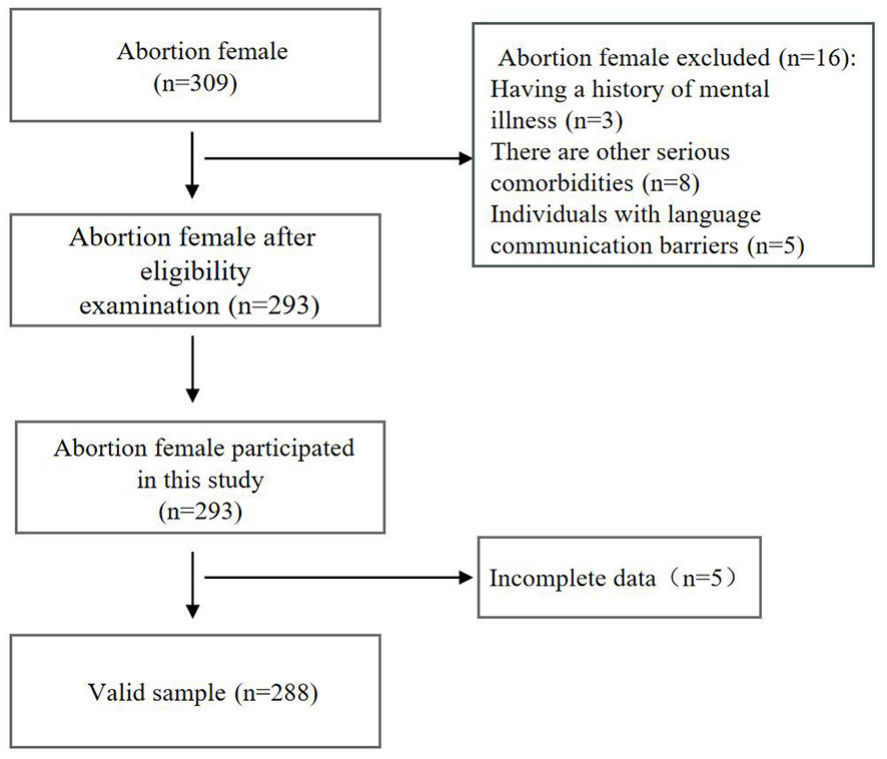
Flowchart of participants selection process.

From December 2024 to November 2025, researchers distributed and collected data from questionnaire survey. Following in-person interviews, the researchers individually filled out the surveys, maintaining the participants' confidentiality. In instances where participants faced difficulties understanding certain items or questions, the researchers provided explanations in a clear and understandable manner on site. Out of 309 women who took part in the survey, 288 completed questionnaires were ultimately gathered. The process of participants selection is detailed in Figure 2. An Excel data table was subsequently set up for two inputters to perform dual data entry, cross-verification, and immediate error correction, thereby ensuring accuracy prior to data analysis.

### 2.3 Sample size

Based on sample size estimation technique (32), the sample size must be at least 5 to 10 times greater than the number of variables. This study comprised a total of 25 variables, including 16 items in the general information questionnaire and 9 scale dimensions. Using the multiplier of 9 for the number of variables, the calculated sample size for this study was 225. Considering a 20.0% attrition rate and invalid scales, a total of 281 participants were required.

### 2.4 Measures

#### 2.4.1 Dependent variable: stigma

In this research, we assessed the stigma faced by participants using the Chinese adaptation of the Individual-Level Abortion Stigma Scale (33). This scale includes four dimensions: external evaluation, loneliness, self-evaluation, and verbal condemnation, consisting of a total of 22 items. The scoring for each item ranges from 0 to 4 points, with higher scores indicating greater levels of stigma. In this research, the Cronbach's α for the scale was measured at 0.90.

#### 2.4.2 Independent variable: family resilience

This research employed the Family Resilience Scale to evaluate the participants' family resilience levels (34). This scale consists of 20 items and is structured into four dimensions: resilience, support, harmony, and openness. The Likert 5-point scoring method was used, resulting in a total possible score of 20 to 100 points. A higher score indicates a stronger level of family resilience. In this research, the Cronbach's α for the scale was measured at 0.96.

#### 2.4.3 Mediator: self-concealment

To assess the degree of self-concealment in women who have had induced abortions, we utilized the Chinese adaptation of the Self Concealing Scale (21). The scale consists of 10 questions and employs a 5-point Likert rating system, yielding a total score range from 10 to 50. A higher score suggests a stronger inclination to hide one's true self. In this research, the Cronbach's α for the scale was measured at 0.90.

#### 2.4.4 Control variables: social-demographic questionnaire

By conducting an extensive review of existing literature and consulting with specialists, a custom-made survey was employed to gather socio-demographic details and control variables from the participants, such as age, permanent residence, marital status, education level, abortion method, reasons for miscarriage, and premarital sexual activity.

### 2.5 Ethical considerations

This research adhered to the principles outlined in the Declaration of Helsinki. This study was approved by the Institutional Review Board of the Deyang people's hospital (Approval No. 202004046). Before the survey, every participant received an informed consent document and had the option to leave the study at any time.

### 2.6 Statistical analysis

IBM SPSS Statistics version 25.0 was employed for the statistical analysis of the data. A two-tailed test with an alpha level of 0.05 was used to assess statistical significance, where *P* < 0.050 were deemed significant. The normality of the data was assessed using the Shapiro Wilk test. In descriptive analysis, normally distributed quantitative data were summarized with means and standard deviations, whereas non-normally distributed data were represented by medians and interquartile ranges. Frequencies and percentages were utilized to present qualitative data. Univariate analysis was conducted to explore factors influencing the stigma of induced abortion using independent sample *t*-test or one-way ANOVA if the data adhered to a normal distribution; otherwise, non-parametric tests were used. In correlation analysis, Pearson's correlation was applied if the data followed a normal distribution, while Spearman's correlation was used for non-normal data. The SPSS PROCESS macro was used to test the mediating model. To evaluate the indirect effect of self-concealment, the bias-corrected (BC) bootstrap method with 5,000 iterations was selected. If the 95.0% BC bootstrap confidence interval (CI) is statistically significant (P < 0.050) and does not encompass zero, it indicates the presence of an indirect effect. The mediation analysis was conducted without adjusting for covariates. Specifically, age, marital status, and educational level—which showed significant associations with stigma in the univariate analyses (Table 1)—were not entered as covariates in the PROCESS model. Therefore, the reported indirect effect should be interpreted as unadjusted for these sociodemographic variables.

**Table 1:** Descriptive statistics (*N* = 288).

Variables	*n*	%	M ±SD (scores of stigma)	*t*/F	*P*
Age					
< 25	72	25.0	53.25 ± 11.34	4.28	0.002^**^
25~29	82	28.5	51.29 ± 12.09		
30~34	76	26.4	49.42 ± 10.87		
35~39	45	15.6	46.13 ± 10.44		
≥40	13	4.5	43.23 ± 11.40		
Marital status					
Unmarried	112	38.9	54.84 ± 10.05	5.84	< 0.001^***^
Married	176	61.1	47.11 ± 11.48		
Educational level					
Junior high school	43	14.9	43.35 ± 8.91	9.18	< 0.001^***^
Senior high school	105	36.5	51.10 ± 9.82		
Undergraduate and above	140	48.6	51.46 ± 12.78		
Abortion method					
Medical abortion	117	40.6	48.55 ± 11.89	−1.92	0.056
Artificial abortion surgery	171	59.4	51.19 ± 11.25		
Causes of miscarriage					
Not using contraceptives	52	18.1	48.44 ± 12.58	2.42	0.090
Contraceptive failure	185	64.2	49.75 ± 11.13		
Medically indicated pregnancy termination	51	17.7	53.16 ± 11.73		
Premarital sex					
Approval	66	22.9	49.03 ± 12.33	1.81	0.165
Not approval	115	39.9	51.70 ± 10.57		
Indifferent	107	37.2	49.08 ± 12.00		
Family resilience (total)			64.08 ± 15.88		
Self-concealment (total)			34.61 ± 7.58		
Stigma (total)			50.12 ± 11.57		

^**^P < 0.010; ^***^P < 0.001; M, mean; SD, standardized difference.

## 3 Results

### 3.1 Descriptive statistics

Table 1 shows the specifics of the descriptive statistics. In this study, 53.0% of women were aged under 30, and the proportion of unmarried women was 38.9%. After normality test, we found that the total score on the stigma was normally distributed and had a uniform variance. The single-variable analysis identified three factors influencing the stigma experienced by women post-abortion: age (F = 4.28, *P* < 0.050). Marital status (*t* = 5.84, *P* < 0.001) and education level (F = 9.18, *P* < 0.001). *Post-hoc* comparisons using the Tukey HSD test were conducted for age and educational level. For age, women younger than 25 years (53.25 ± 11.34) had significantly higher stigma scores than those aged 35–39 years (46.13 ± 10.44, *P* = 0.009) and those aged 40 years or older (43.23 ± 11.40, *P* = 0.029). No significant differences were found between other age groups (all *P* > 0.05). For educational level, women with junior high school education (43.35 ± 8.91) had significantly lower stigma scores than those with senior high school (51.10 ± 9.82, *P* = 0.001) and those with undergraduate or above (51.46 ± 12.78*, P* < 0.001). No significant difference was observed between the senior high school and undergraduate or above groups (P = 0.968). Other variables, including abortion method (*t* = −1.916, *P* = 0.056), reasons for miscarriage (F = 2.424, *P* = 0.090), and premarital sex attitudes (F = 1.812, *P* = 0.165), were not significantly associated with stigma. All descriptive data (means, standard deviations, frequencies, and percentages) are presented in Table 1.

### 3.2 Correlations among family resilience, self-concealment, and stigma

The mean scores of stigma, family resilience, and self-concealment were 50.12 ± 11.57. 64.08 ± 15.88 and 34.61 ± 7.58. respectively. Family resilience was negatively correlated with self-concealment (r = −0.390, *P* < 0.010) and stigma (r = −0.491, *P* < 0.010). Whereas self-concealment was positively correlated with stigma (r = 0.444, *P* < 0.010) (Table 2).

**Table 2:** Correlations among family resilience, self-concealment and stigma.

Variables	Family resilience	Self-concealment	Stigma
Family resilience	1		
Self-concealment	−0.39^**^	1	
Stigma	−0.49^**^	0.44^**^	1

^**^P < 0.010.

### 3.3 Mediating effect of self-concealment

**Figure 3:**
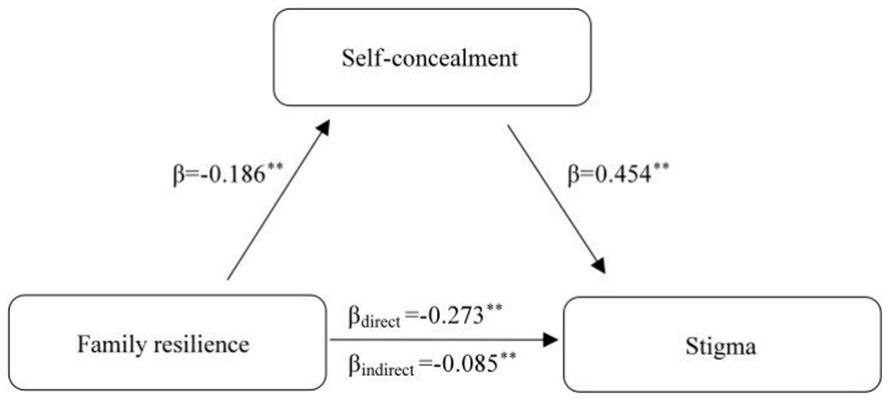
Theoretical model of this study. Mediating effect of self-concealment on the relationship between family resilience and stigma. **P < 0.01.

According to Table 3, there was a notable negative correlation between family resilience and both stigma (β = −0.27, *P* < 0.001) and self-concealment (β = −0.19, *P* < 0.001). Self-concealment mediated the relationship between family resilience and stigma (β =0.45, *P* < 0.001). Family resilience had a direct impact of −0.27, with a 95.0% confidence interval adjusted for bootstrap bias ranging from −0.13 to −0.05. The total effect of family resilience on stigma through self-concealment was −0.36 (Figure 3).

**Table 3:** Mediating effect of self-concealment on the relationships between family resilience and stigma.

Indirect effect	Effect of X on M	Effect of M on Y	Direct effect	Indirect effect	Total effect	Effect size	Bootstrapping (BC 95% CI)
Family resilience → self-concealment → stigma	–0.186^***^	0.454^***^	–0.273^***^	–0.085	–0.358	23.46%	–0.127, –0.048

^***^P < 0.001; X, independent variable (family resilience); M, mediator (self-concealment); Y, dependent variable (stigma); CI, confidence interval; BC, bias-corrected.

## 4 Discussion

### 4.1 Summary of main results

This research found that women who had experienced induced abortion had an average stigma score of 50.12 ± 11.57. Suggesting that the stigma level was moderately high. The level of stigma was found to differ according to marital status, age, and educational level. Moreover, our findings indicated that increased family resilience was correlated with reduced stigma, with self-concealment potentially playing a partial mediating role in this relationship, which is consistent with our hypotheses.

### 4.2 Sociodemographic variables associated with stigma

Studies have suggested that unmarried women, compared to married ones, frequently manifested higher psycho-psychological issues following an unintended pregnancy (35, 36). In this study, we also found that unmarried women exhibited higher levels of stigma compared to married women. Our study found that 40.0% of women held negative views toward premarital sex, suggesting that it is not entirely accepted by society, or that acceptance levels are low. In particular, some parents of girls consider premarital sex as “shameful” and impart the belief that women engaging in premarital sex may be branded as “immoral” and could face public opinion pressure and moral critique (35, 37, 38). In such a social environment, unmarried women are more susceptible to experiencing a higher level of stigma.

In line with earlier findings (36), this research revealed a link between younger age and heightened stigma experienced by women who have undergone abortions. One possible reason may be that the underdeveloped psychological resilience of younger women, which may hinder their ability to appropriately cope with external pressures such as stigma and discrimination during crisis situations (36). Furthermore, we discovered that individual with higher education levels experienced greater stigma. This might be due to the reality that women with advanced education frequently possess a well-developed social circle, making them more sensitive to societal judgments and peer discussions (39). As a result, they might hide their circumstances to prevent external stigma, which in turn may increasetheir self-stigma (39). Consistent with these findings, studies conducted in Türkiye, a country with similar conservative norms regarding gender and reproduction, have also reported that sociodemographic factors such as education level, marital status, and socioeconomic status are significantly associated with abortion stigma (40, 41).

### 4.3 Correlations among stigma, self-concealment, and family resilience

The research demonstrated a notable inverse relationship between family resilience and stigma, showing that increased family resilience was associated with lower stigma levels, consistent with earlier findings (42). Earlier research indicates that women who have undergone abortions experience significant stress due to factors like environmental pressure (43, 44). The family unit plays a crucial role in alleviating these stressors and may have has a beneficial effect on individuals (45, 46). On the other hand, women who have undergone abortions and possess lower family resilience might struggle with poor communication within their families, leading to increased self-isolation and feelings of being misunderstood or undervalued, which may heighten self-stigma (46, 47).

Moreover, this study revealed that self-concealment among women who have had induced abortions may play a partial mediating role between family resilience and stigma. This suggests that the family resilience of these women may be linked to their level of stigma through self-concealment. However, the process of self-concealment can also be a source of stress, and concealing an induced abortion is often a negative coping strategy that hinders individuals from rationally interpreting their experiences during stressful event (4). On the other hand, when women with high family resilience receive proactive support and encouragement from their family members, it may help reduce their psychological distress, promote and constructive cognitive processing, and encourage them to disclose pressures to others (45, 47).

### 4.4 Implications

This research showed that family resilience may be associated with reduced stigma among women who have undergone abortions, both directly and indirectly, offering a theoretical foundation for creating specific interventions. In China, discussions about abortions are infrequent between women and their parents, which hinders the receipt of family support and may lead to increased self-stigma (48). Thus, interventions toward enhancing family resilience should be culture-specific in China. To collectively mitigate the adverse effects of abortion, it is essential to enhance family dialogue, ensuring efficient information sharing, balanced emotional expression, and emotional bonding between women who have undergone abortions and their relatives (49). Moreover, medical practitioners can create tailored psychological support plans to help women who have undergone abortions lower their tendency to hide their experiences, foster positive health attitudes, and boost their stress management confidence, thereby encouraging proper comprehension and coping.

### 4.5 Limitations

Several limitations of this study should be acknowledged. First, the cross-sectional design precludes the establishment of temporal or causal relationships among family resilience, self-concealment, and stigma. Although we proposed a mediation model based on psychological stress theory, the direction of effects cannot be determined. Alternative models (e.g., stigma influencing family resilience, or self-concealment affecting family resilience) cannot be ruled out. Therefore, the mediation results presented in this study should be interpreted as exploratory and association-based, rather than causal. Longitudinal or experimental studies are needed to clarify the temporal sequence and confirm the proposed mediating role of self-concealment. Second, this study employed convenience sampling and recruited participants from a single public hospital in Sichuan Province, China. This limits the generalizability of the findings to other populations, geographic regions, or healthcare settings. Women from different cultural backgrounds, socioeconomic statuses, or with different abortion experiences may exhibit different levels of stigma, family resilience, and self-concealment. Selection bias may also be present, as participants who agreed to join the study might differ from those who declined. Therefore, future multicenter studies with probability sampling are needed to validate and extend our results. Third, as noted in the Methods section, the mediation analysis did not adjust for sociodemographic variables (age, marital status, and educational level) that were significantly associated with stigma in the univariate analyses (Table 1). This lack of adjustment may introduce confounding bias, and the observed indirect effect should be interpreted with caution. Future studies with larger sample sizes should include these variables as covariates to test the robustness of the mediating effect. Fourth, the use of self-report questionnaires may introduce recall and social desirability bias, particularly for sensitive topics such as abortion. Despite these limitations, this study provides valuable insights into the relationships among family resilience, self-concealment, and stigma in Chinese women who have had induced abortions.

## 5 Conclusion

The research indicated that women who underwent abortions faced stigma levels higher than moderate, potentially associated with their age, marital status, and education. Moreover, the research indicated that family resilience is inversely related to stigma, with self-concealment potentially acting as a mediator between family resilience and stigma. However, due to the cross-sectional design, these findings should be interpreted as associations rather than causal relationships. Healthcare providers ought to prioritize the familial context of women who have undergone abortions and create interventions that are culturally appropriate. To further confirm the results of this research and enhance the wellbeing of women who have undergone abortions from personal, familial, and societal viewpoints, future longitudinal and intervention-based studies are essential.

## Data Availability

The original contributions presented in the study are included in the article/Supplementary material, further inquiries can be directed to the corresponding author.
